# Bowel ultrasound for predicting surgical management of necrotizing enterocolitis: a systematic review and meta-analysis

**DOI:** 10.1007/s00247-017-4056-x

**Published:** 2017-12-19

**Authors:** Alain C. Cuna, Nidhi Reddy, Amie L. Robinson, Sherwin S. Chan

**Affiliations:** 1 Department of Pediatrics, Division of Neonatology, Children’s Mercy Kansas City, Kansas City, MO USA; 2 School of Medicine, University of Missouri-Kansas City, Kansas City, MO USA; 3 Department of Radiology, Children’s Mercy Kansas City, 2401 Gillham Road, Kansas City, MO 64108 USA

**Keywords:** Bowel, Gastrointestinal tract, Infants, Necrotizing enterocolitis, Ultrasound

## Abstract

**Background:**

Necrotizing enterocolitis (NEC) is a devastating inflammatory disease of the intestinal tract that represents a significant source of morbidity and mortality in preterm infants. Imaging of the abdomen is valuable for timely diagnosis and close monitoring of disease progression in NEC. Bowel ultrasound (US) is increasingly being recognized as an important imaging tool for evaluating NEC that provides additional detail than plain abdominal radiographs.

**Objective:**

To identify bowel US findings associated with surgical management or death in infants with NEC.

**Materials and methods:**

We searched Embase, PubMed, and the Cumulative Index to Nursing and Allied Health Literature for studies investigating the association between bowel US findings and surgical management or death in NEC. Selected articles were evaluated for quality of study methodology using the Newcastle-Ottawa Scale, and aggregate statistics for odds ratio (OR) and 95% confidence interval were calculated.

**Results:**

Of 521 articles reviewed, 11 articles comprising 748 infants were evaluated for quality. Nine of the studies were retrospective and from single-center experiences. Pooled analysis showed that focal fluid collections (OR 17.9, 3.1–103.3), complex ascites (OR 11.3, 4.2–30.0), absent peristalsis (OR 10.7, 1.7–69.0), pneumoperitoneum (OR 9.6, 1.7–56.3), bowel wall echogenicity (OR 8.6, 3.4–21.5), bowel wall thinning (OR 7.11.6–32.3), absent perfusion (OR 7.0, 2.1–23.8), bowel wall thickening (OR 3.9, 2.4–6.1) and dilated bowel (OR 3.5, 1.8–6.8) were associated with surgery or death in NEC. In contrast, portal venous gas (OR 3.0, 0.8–10.6), pneumatosis intestinalis (OR 2.1, 0.9–5.1), increased bowel perfusion (OR 2.6, 0.6–11.1) and simple ascites (OR 0.54, 0.1–2.5) were not associated with surgery or death.

**Conclusion:**

This meta-analysis identified several bowel US findings that are associated and not associated with surgery or death in NEC. Bowel US may be useful for early identification of high-risk infants with NEC who may benefit from more aggressive treatment, including surgery. Future studies are needed to determine whether the addition of bowel US in NEC evaluation would improve outcomes.

**Electronic supplementary material:**

The online version of this article (10.1007/s00247-017-4056-x) contains supplementary material, which is available to authorized users.

## Introduction

Necrotizing enterocolitis (NEC) represents a significant cause of neonatal morbidity and mortality especially in preterm infants. It is estimated that about 5% to 14% of infants weighing <1500 g at birth develop NEC and about 25% to 40% die because of it [1–3]. Because NEC can rapidly progress to intestinal perforation, peritonitis and shock, timely diagnosis and treatment are essential for better outcomes [4]. Diagnosis of NEC is made based on clinical presentation, laboratory tests and imaging. The role of imaging is important to confirm the diagnosis and follow disease progression, but interpretation can also be challenging as normal or nonspecific findings do not rule out NEC entirely [5]. Initial treatment – which involves bowel rest, intravenous fluids, broad-spectrum antibiotics and other supportive measures – can be ineffective in controlling disease progression in some infants, and surgical intervention with either peritoneal drainage or bowel resection may be required [6, 7]. The transition from medical to surgical care represents a critical decision-making point in NEC management that can have important implications for clinical outcomes.

Traditionally, plain abdominal radiography is the imaging modality of choice for evaluating infants for NEC [8]. However, abdominal radiographs are challenging to interpret due to wide variability and significant overlap between radiographic signs of NEC and other intestinal pathology [9]. Abdominal radiographs are also limited in characterizing disease progression; free air, which radiographs can detect reasonably well, is already a late finding that indicates intestinal perforation. There is also lack of evidence regarding optimal frequency, use of one versus two views, and frequency and duration of radiographic imaging for monitoring disease progression in NEC.

Bowel ultrasound (US) has been reported as a useful adjunct to radiography for evaluating NEC [9, 10]. Advantages of bowel US include real-time assessment of peristalsis, vascular perfusion, bowel wall thickening and abdominal fluid [11]. Small, observational studies suggest that additional details provided by bowel US may be helpful for earlier identification of high-risk infants with NEC who may benefit from more aggressive treatment, including surgery. It is unclear from the literature, however, which bowel US findings provide the most useful prognostic information to help guide clinical decision-making. This systematic review and meta-analysis summarize evidence from existing literature and identify bowel US findings associated with surgical management or death in infants with NEC.

## Materials and methods

This systematic review was reported in accordance with the Preferred Reporting Items for Systematic Reviews and Meta-Analyses (PRISMA) statements and an ethical review was not required. The protocol for this systematic review was registered on PROSPERO (doi: CRD42017068467) and is available in full on its website (www.crd.york.ac.uk/PROSPERO/display_record.asp?ID=CRD42017068467).

### Search strategy

A literature review of Embase, PubMed, and the Cumulative Index to Nursing and Allied Health Literature (CINAHL) was conducted up to December 2016. We used the keywords “necrotizing or necrotizing enterocolitis” and “ultrasound” (including variations such as “ultrasonography,” “sonograph,” etc.). Complete details of the search strategy are available in the supplement (Online Resource 1). A search of references cited in review articles and selected articles of interest was also done, and an updated search encompassing January to June 2017 captured additional studies that may have been published since the original search.

### Study selection criteria

A neonatologist (A.C.C., 3 years of experience) and senior medical student (N.R.) performed the literature search. Articles identified through the literature search were screened by reading through their title and abstract. Studies were included if they investigated the association of bowel US in predicting the outcome of surgical management or death in infants with NEC. Surgical management was defined as either laparotomy or placement of peritoneal drain, and death was defined to include those resulting from NEC or all other causes. Full-text manuscripts were obtained for the articles that met these inclusion criteria. Case reports, review articles, meeting abstracts and letters to the editor were excluded. Disagreements about study eligibility were resolved via discussion with the senior author (S.S.C., pediatric radiologist with 3 years of experience).

### Data extraction and quality assessment

Two authors (A.C.C. and N.R.) independently extracted and tabulated the following data from each study: study characteristics, including study design and sample size; patient characteristics, including gestational age, birth weight, gender and diagnosis of NEC; treatment outcome, including medical treatment, surgical treatment and death, and bowel US findings assessed to predict outcome. Differences in collected data were resolved by discussion with the senior author (S.S.C.). Authors of studies were contacted in cases where relevant data for analysis were missing or difficult to ascertain from the manuscript.

The methodological quality of included studies was assessed using the Newcastle-Ottawa Scale [12], which is specifically designed to assess quality of non-randomized studies in meta-analysis. Studies were assessed based on cohort selection, comparability and outcome assessment using nine multiple-choice questions. Studies were deemed of good quality if the total score was 7 or higher, of fair quality if the total score was 6 and of poor quality if the score was 5 or lower. In cases of disagreements, consensus was reached by discussion.

### Data analysis

For each article, 2 × 2 tables were created to assess the association between individual bowel US imaging characteristics and outcome of either surgical management alone or combined outcome of surgery or death. Bowel US findings evaluated by three or more studies were included for analysis. Pooled odds ratio (OR) estimates and 95% confidence interval (CI) were calculated to measure the strength of association between various bowel US findings and the outcomes of interest. Statistical analysis was performed using Review Manager (RevMan Version 5.3; The Nordic Cochrane Centre, Copenhagen, Denmark) with statistical significance set at *P*<0.05. Study heterogeneity was assessed using the inconsistency (I^2^) test, with an I^2^ estimate greater than or equal to 50% considered as having substantial heterogeneity.

## Results

**Fig. 1:**
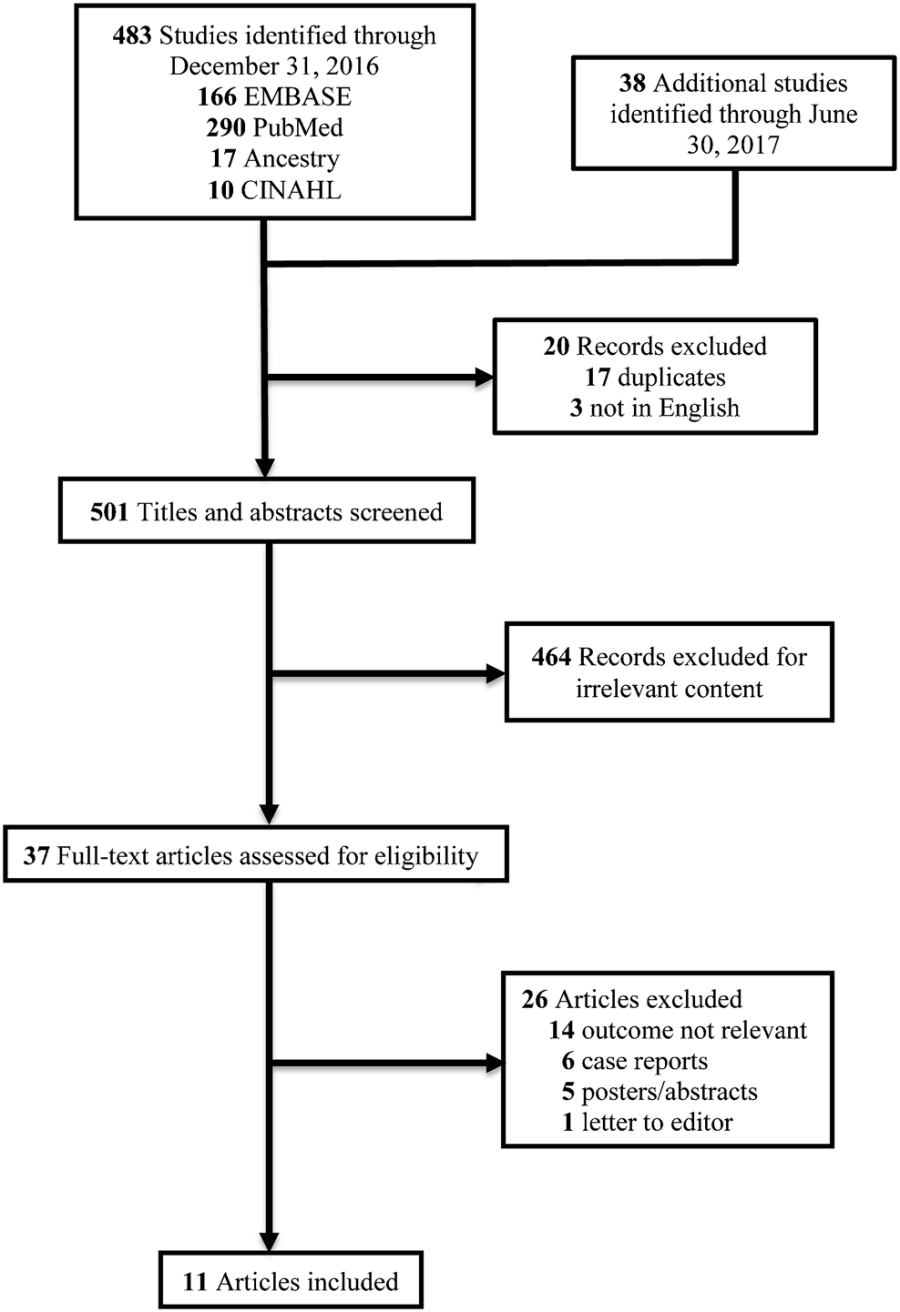
Flow diagram of study selection

Our systematic literature search found 521 articles. Of these, 11 articles reporting on 748 infants met eligibility criteria and were included in our final analysis (Fig. 1) [13–23]. The characteristics of the included studies are summarized in Table 1. The gestational age range of the infants was 23 to 41 weeks. Two of the studies were prospective cohorts, six were retrospective cohorts and three were case series. Bowel US was performed as part of routine evaluation for NEC in five studies, while the remaining six studies performed bowel US selectively per clinical discretion. In seven studies, bowel US was performed in infants with suspected NEC (Bell’s stage ≥1), while in four studies bowel US was performed on infants with definite NEC (Bell’s stage ≥2). The outcome of surgery or death was evaluated in seven studies, while four studies evaluated the outcome of surgical management only.

**Table 1:** Characteristics of included studies on ultrasound of necrotizing enterocolitis (NEC)

Study	Country	Study design	Sample size	Demographic characteristics	Type of patients	Indication for bowel US	Outcomes
Lindley et al. 1986 [13]	USA	CS	15	GA mean: 33 wks BW mean: 1705 g	Suspected NEC	Selective	Surgery
Bomelburg et al. 1992 [14]	Germany	CS	27	GA not recorded BW range: 700–4010 g	Suspected NEC	Selective	Surgery
Silva et al. 2007 [15]	Canada	RC	40	GA medical group mean: 31 wks (range: 24–40); surgery/death group mean: 29 wks (range: 24–40) BW medical group mean: 1546 g (range: 680–3774); surgery/death group mean: 1361 g (range: 540–3500)	Definite NEC	Selective	Surgery or death
Muchantef et al. 2013 [16]	USA	RC	44	GA medical group mean: 31 wks (range: 26–40); surgery/death group mean: 31 wks (range: 24–40) BW medical group mean: 1480 g (range: 900–2100); surgery/death group mean: 1360 g (range: 700–3260)	Suspected NEC	Selective	Surgery or death
Garbi-Goutel et al. 2014 [17]	France	RC	95	GA mean: 28.7±2 wks BW mean: 1143±311 g	Suspected NEC	Routine	Surgery or death
Yikilmaz et al. 2014 [18]	Canada	PC	26	GA median: 29 wks (range: 24–41) BW median: 1350 g (range: 730–3420)	Definite NEC	Routine	Surgery
Staryszak et al. 2015 [19]	Poland	CS	9	GA range: 24–40 wks BW range: 540–2600 g	Suspected NEC	Selective	Surgery or death
Prithviraj et al. 2015 [20]	India	PC	60	GA mean: 30.5±0.5 wks (range: 25–36) BW mean: 1500±201 g (range: 600–2400)	Suspected NEC	Routine	Surgery or death
He et al. 2016 [21]	China	RC	238	GA medical group median: 32 wks (IQR: 30–33); surgery group median: 32 (IQR: 29–33) BW medical group median: 1800 g (IQR: 1450–2320); surgery group median: 1960 g (IQR: 1432–2400)	Definite NEC	Routine	Surgery
Wang et al. 2016 [22]	China	RC	144	GA mean: 33±3 wks (range: 25–40) BW mean: 2045±798 g (range: 740–4410)	Suspected NEC	Routine	Surgery or death
Palleri et al. 2017 [23]	Sweden	RC	25	GA median: 25.6 wks (range: 23–35) BW median: 824 g (range: 545–3200)	Definite NEC	Selective	Surgery or death

*BW* birthweight, *CS* case series, *g* grams, *GA* gestational age, *IQR* interquartile range, *NEC* necrotizing enterocolitis*, PC* prospective cohort, *RC* retrospective cohort, *wks* weeks

### Quality assessment

Quality assessment of the included studies is summarized in Table 2. All studies scored 6 to 7 out of a possible 9 on the Newcastle-Ottawa Scale, indicating fair to good quality studies. The criterion most studies scored poorly on was “comparability,” which examines for adjustment of important confounders in the analysis.

**Table 2:** Quality assessment of selected studies

Study	Selection	Comparability	Outcome	Total score	Quality
Lindley et al. 1986 [13]	* * * -	–	* * *	6	Fair
Bomelburg et al. 1992 [14]	* * * -	–	* * *	6	Fair
Silva et al. 2007 [15]	* * * *	–	* * *	7	Good
Muchantef et al. 2013 [16]	* * * *	–	* * *	7	Good
Garbi-Goutel et al. 2014 [17]	* * * *	–	* * *	7	Good
Yikilmaz et al. 2014 [18]	* * * *	–	* * *	7	Good
Staryszak et al. 2015 [19]	* * * -	–	* * *	6	Fair
Prithviraj et al. 2015 [20]	* * * *	–	* * *	7	Good
He et al. 2016 [21]	* * * *	–	* * *	7	Good
Wang et al. 2016 [22]	* * * *	–	* * *	7	Good
Palleri et al. 2017 [23]	* * * *	–	* * *	7	Good

Each asterisk (*) and dash (−) indicates whether individual criterion within each subsection of Newcastle-Ottawa Score was fulfilled or not fulfilled, respectively. Total score reflects total number of fulfilled criteria

### Pooled analyses

Thirteen bowel US findings were evaluated by at least three studies and were included in the meta-analysis. Included studies did not have any missing or unclear data that required contacting study authors. Decreased bowel perfusion, peritoneal calcification, increased superior mesenteric artery flow, and reduced inflation of the intestine were each evaluated only once and were excluded. Decreased peristalsis and ascites (unspecified if simple or complex) were evaluated twice and were also excluded. One study [19] evaluated decreased or absent peristalsis together, while another study [21] assessed complex ascites and focal fluid collections together (both studies were also excluded from further analysis). Studies that investigated the association of bowel US findings with surgery alone were analyzed separately from studies that investigated the combined outcome of surgery or death. Bowel US findings associated with poor outcomes in NEC are summarized in Table 3, while bowel US findings not associated with poor outcomes in NEC are summarized in Table 4. Forest plots summarizing the association of various bowel US findings with surgery or death are also provided in the supplement (Online Resource 2–14).

**Table 3:** Bowel US findings associated with surgery or death in necrotizing enterocolitis

Bowel US finding	Number of studies	Number of patients	Odds ratio	95% confidence interval	*P*-value
Pneumatosis					
Surgery	3	287	2.23	1.01–4.92	0.05
Pneumoperitoneum					
Surgery or death	6	417	9.63	1.65–56.32	0.01
Bowel wall echogenicity					
Surgery or death	3	104	8.58	3.42–21.53	<0.01
Bowel wall thickening					
Surgery	2	264	4.74	2.53–8.89	<0.01
Surgery or death	7	442	3.86	2.43–6.14	<0.01
Bowel wall thinning					
Surgery or death	5	189	7.11	1.56–32.29	0.01
Absent perfusion					
Surgery or death	3	120	6.99	2.06–23.76	0.002
Absent peristalsis					
Surgery or death	4	298	10.68	1.65–69.02	0.01
Complex ascites					
Surgery or death	3	104	11.28	4.23–30.04	<0.01
Focal fluid collection					
Surgery or death	5	275	17.92	3.11–103.31	0.001
Dilated bowel					
Surgery or death	3	222	3.50	1.81–6.75	<0.01

**Table 4:** Bowel US findings not associated with surgery or death in necrotizing enterocolitis

Bowel US finding	Number of studies	Number of patients	Odds ratio	95% confidence interval	*P*-value
Portal venous gas					
Surgery	4	306	1.94	0.99–3.77	0.05
Surgery or death	7	442	2.98	0.84–10.60	0.09
Pneumatosis					
Surgery or death	7	442	2.07	0.85–5.05	0.11
Bowel wall thinning					
Surgery	2	264	3.01	0.88–10.27	0.08
Increased perfusion					
Surgery or death	4	180	2.60	0.61–11.13	0.20
Simple ascites					
Surgery	2	264	0.46	0.03–6.97	0.58
Surgery or death	5	189	0.54	0.12–2.47	0.43

### Portal venous gas, pneumatosis intestinalis and free air

Portal venous gas and pneumatosis intestinalis were evaluated in 11 and 10 studies, respectively, while free air was evaluated in six studies. Pooled analysis showed that portal venous gas was not associated with either surgery (OR 1.94, 95% CI 0.99–3.77) or the combined outcome of surgery or death (OR 2.98, 95% CI 0.84–10.60). Pneumatosis was also not associated with surgery or death (OR 2.07, 95% CI 0.85–5.05) but was associated with surgery (OR 2.23, 95% CI 1.01–4.92). As expected, free air was found to be strongly associated with surgery or death (OR 9.63, 95% CI 1.65–56.32) in pooled analysis.

### Bowel wall: thickening, thinning, and echogenicity

Bowel wall thickening was evaluated in nine studies, while bowel wall thinning was included in seven studies. The cutoff measurements used for bowel wall thickening and thinning were 2.6 mm and 1.0 mm, respectively, although two studies [21, 22] did not provide clear definitions. Pooled analysis revealed that bowel wall thickening, but not bowel wall thinning, was associated with increased risk for surgery (bowel wall thickening: OR 4.74, 95% CI 2.53–8.89 and bowel wall thinning: OR 3.01, 95% CI 0.88–10.27). When looking at studies that investigated surgery or death, pooled analysis showed both bowel wall thickening and thinning were associated with this combined outcome (bowel wall thickening: OR 3.86, 95% CI 2.43–6.14 and bowel wall thinning: OR 7.11, 95% CI 1.56–32.29). An increase in bowel wall echogenicity, which was defined as an increase in mural echogenicity with loss of hypoechogenic muscle layer [9], was assessed in three studies and found to be associated with an increased risk for surgery or death (OR 8.58, 95% CI 3.42–21.53).

### Peristalsis

Five studies looked at absent peristalsis in NEC. No studies specified whether absent peristalsis was localized or generalized throughout the bowel. One study [21] evaluated its association with surgery and was excluded from further analysis. The remaining four studies evaluated surgery or death as a combined outcome and found it to be associated with absent peristalsis on bowel US (OR 10.68, 95% CI 1.65–69.02).

### Bowel perfusion

Five studies assessed the association of increased bowel perfusion using color Doppler with NEC outcomes, although increased perfusion was variably defined. Two studies [15, 20] objectively defined increased perfusion as having more than nine dots of color Doppler signal per square centimeter; another two studies [16, 23] used more subjective criteria based on patterns of flow and one study [18] did not provide a clear definition for increased perfusion. No studies evaluated bowel perfusion using Doppler waveforms. Only one study [18] investigated increased perfusion with outcome of surgery and was excluded from further analysis. The remaining four studies investigated the combined outcome of surgery or death. Pooled analysis of these four studies showed that increase in bowel perfusion was not associated with surgery or death in NEC (OR 2.60, 95% CI 0.61–11.13). Absent perfusion was assessed in four studies. Of these, we pooled analysis from the three studies that assessed the outcome of surgery or death. In contrast to increased perfusion, we found that absent perfusion was associated with the combined outcome of surgery or death (OR 6.99, 95% CI 2.06–23.76).

### Abdominal fluid: simple ascites, complex ascites, and focal fluid collections

Seven studies investigated the association of simple ascites with outcomes in NEC – two studies looked at surgery alone, while five studies looked at surgery or death. Pooled analysis showed that simple ascites was not associated with either surgery alone (OR 0.46, 95% CI 0.03–6.97) or surgery or death (OR 0.54, 95% CI 0.12–2.47). Of the four studies that evaluated complex ascites, only three studies that assessed surgery or death were pooled for analysis. In contrast to simple ascites, complex ascites was found to be associated with surgery or death (OR 11.28, 95% CI 4.23–30.04). Six studies assessed the association of focal fluid collection with outcomes in NEC. One study [18] evaluated for the outcome of surgery, while the remaining five studies evaluated for the combined outcome of surgery or death. Pooled analysis from these five studies showed that focal fluid collection was associated with surgery or death (OR 17.92, 95% CI 3.11–103.31).

### Intestinal dilation

Intestinal dilation was not clearly defined by the three studies that assessed its association with surgery or death. Nevertheless, its presence was found to be associated with an increased risk for surgery or death (OR 3.50, 95% CI 1.81–6.75).

## Discussion

In this meta-analysis, we identified several bowel US characteristics associated with poor outcomes in infants with NEC. Bowel US features most strongly associated with surgery or death were free air, absent peristalsis, complex ascites and focal fluid collection. Other features with more moderate association with surgery or death included bowel wall thinning, increased bowel wall echogenicity, bowel wall thickening, absent perfusion and dilated bowel. We also identified bowel US findings not associated with surgical NEC or death, including portal venous gas, increased bowel perfusion and simple ascites. Together, these findings suggest that the addition of bowel US in evaluating infants with NEC may help identify infants at high-risk for poor outcomes.

The current standard for imaging evaluation of infants with suspected NEC is plain abdominal radiography. However, the use of US as an imaging adjunct to radiography has increased in recent years. Initial case reports from the 1980s described the ability of US to detect pneumatosis and portal venous gas in infants with NEC [24–26]. Since then, use of US in NEC has extended to include evaluation of bowel wall, bowel perfusion and abdominal fluid [9]. Bowel US has been used as an adjunctive imaging tool for diagnosis in infants with clinical suspicion for NEC but whose plain abdominal radiographs show nonspecific findings [27]. Bowel US has also been used for follow-up of infants already diagnosed with NEC to monitor disease progression and guide clinicians regarding management, including surgery [9, 28]. The majority of these studies, however, were small single-center studies. In this systematic review, we combined data from these different studies in a meta-analysis and identified bowel US findings associated with surgery or death. These bowel US findings may provide valuable prognostic information and can be used to support more aggressive management including surgery.

Of the 13 bowel US findings included in this review, only 3 – free air, pneumatosis and portal venous gas – can also be accurately assessed by plain radiography. Although other findings – including ascites, bowel wall thickening and nonviable bowel – can be insinuated from serial radiographs, bowel US is more capable of characterizing these findings in greater detail. We found that US evidence of free air, but not of pneumatosis or portal venous gas, was associated with increased risk of surgery or death. Pneumatosis was also associated with surgery, while portal venous gas was not – although confidence interval values indicated a trend toward positive association. Some studies have suggested that portal venous gas was associated with worse outcomes including need for surgery [29, 30], although a more recent prospective study found that clinical severity is more important than portal venous gas in predicting poor outcomes [31]. Taken together, these results are consistent with current clinical practice regarding radiographs in NEC and likely reflect similar interpretation given to portal venous gas, pneumatosis and free air whether demonstrated on bowel US or radiographs.

Several bowel US features used to evaluate NEC included assessment of the bowel wall. The seminal study by Faingold et al. [28], in which bowel US was performed in 30 neonates with proven or suspected NEC and 30 normal neonates as controls, provided reference values for normal bowel wall thickness and echogenicity that have been widely used in other studies. Faingold et al. [28] also performed color Doppler interrogation of the bowel wall and assessed bowel perfusion as normal, increased or absent perfusion based on subjective assessment of flow patterns and objective measurement of color Doppler signals or “dots” per square centimeter. In this review, bowel wall thickening and thinning, increased bowel wall echogenicity, absent perfusion and absent peristalsis were all associated with surgery or death of infants with NEC. These bowel wall findings are thought to represent a continuum of changes that reflects worsening severity of NEC [9]. In the initial stages, bowel wall thickness, echogenicity and perfusion are increased due to ongoing intestinal inflammation and mucosal edema. As NEC progresses, bowel wall thinning, absent perfusion and absent peristalsis become more predominant and are highly suggestive of impending bowel perforation. The dynamic changes in bowel wall findings, which cannot be adequately evaluated by radiography, provide a distinct advantage for bowel US in characterizing disease progression of NEC before perforation occurs.

US is also superior to radiography in characterizing fluid in the abdomen. In this review, US findings of complex ascites and focal fluid collections were associated with surgery or death, while simple (anechoic) ascites was not. This is consistent with other studies that found that free, anechoic intraperitoneal fluid can be a normal finding in neonates [28], whereas ascitic fluid with echoes or septations is more suggestive of pus or intestinal contents that indicate perforation [32, 33].

It is important to note that careful consideration of imaging together with clinical and laboratory findings is important in assessing infants for NEC. For example, although positive findings in imaging such as portal venous gas or pneumatosis are strongly suggestive of NEC, other possibilities such as inadvertent injection of air through an umbilical venous catheter [34] or cow milk protein allergy [35] should also be considered, especially when clinical and laboratory findings are otherwise reassuring. Likewise, a normal or equivocal imaging finding by itself cannot rule out NEC entirely, especially when clinical and laboratory findings are strongly suggestive of it.

This meta-analysis has several limitations. The majority of included studies were retrospective observational cohorts, which may overestimate the strength of association between US findings and outcomes in NEC. The wide confidence intervals in some point estimates also suggest small sample size and lack of homogeneity across studies. Publication bias, where only positive studies are reported in the literature, could also have influenced the results of this review, although we sought to systematically search the gray literature for unpublished studies. There was also considerable variation among the different studies. As a result, we were often only able to analyze data from at most seven studies at once, despite identifying 11 studies of interest. Some studies reported on bowel US performed in infants with suspected NEC, while others included only infants with definite NEC (Modified Bell’s stage 2–3). In some studies, bowel US was part of routine evaluation for NEC, while in others bowel US was used selectively per clinical discretion. Although variation among the different studies is an important limitation and potential source of bias, such differences also closely reflect clinical practice. Indications for surgical intervention were also not clearly delineated and likely varied between studies. A potential bias exists in that US results may have contributed to decisions to perform surgery. Comparison to objective surgical or pathological findings was not possible because these findings were not reported in the source studies. Studies were also conducted over the course of 30 years during which US capability has significantly changed, with more recent machines capable of higher spatial and contrast resolution and cine captures. Included studies also analyzed the association of each bowel US finding with surgery or death separately. This does not reflect clinical practice where US findings are analyzed and interpreted together. Thus, the prognostic value of one bowel US finding by itself, or of two or more bowel US findings combined, cannot be determined.

## Conclusion

This meta-analysis identified several bowel US findings associated with surgery or death in necrotizing enterocolitis including free air, absent peristalsis, absent perfusion, complex ascites, focal fluid collection, bowel wall thinning and thickening, increased bowel wall echogenicity and dilated bowel. We also identified bowel US findings not associated with surgical necrotizing enterocolitis or death, such as portal venous gas, increased bowel perfusion and simple ascites. We conclude that bowel US may be used for early identification of high-risk infants with necrotizing enterocolitis who may benefit from more aggressive treatment, including surgery. Future studies are needed to determine (1) the optimal timing and frequency of bowel US that would be most useful in the diagnostic pathway for necrotizing enterocolitis and (2) whether the addition of bowel US in necrotizing enterocolitis evaluation would improve outcomes.

## Electronic supplementary material

ESM 1 (PDF 2.67 mb)
