# Supplementary material for: Bowel ultrasound for predicting surgical management of necrotizing enterocolitis: a systematic review and meta-analysis
Source: Pediatr Radiol. 2017 Dec 19;48(5):658–66. doi: 10.1007/s00247-017-4056-x (PMC5895673; doi:10.1007/s00247-017-4056-x)
Supplement: Supplementary file 1 — (PDF 2.67 mb) [file 247_2017_4056_MOESM1_ESM.pdf]

Pubmed search:
("Enterocolitis, Necrotizing"[Mesh] OR "necrotizing enterocolitis" OR "necrotising enterocolitis") AND ("Ultrasonography"[Mesh] OR ultrasonograph* OR sonograph* OR ultrasound*) AND (English[lang])
CINAHL search:
((MH “Enterocolitis, Necrotizing") OR "necrotizing enterocolitis" OR "necrotising enterocolitis") AND ((MH"Ultrasonography") OR ultrasonograph* OR sonograph* OR ultrasound*) AND Limiters – English Language, Expanders – Apply related words; Also search within the full text of the articles; Apply equivalent subjects
Embase search:
(‘necrotizing enterocolitis’/exp OR ‘necrotizing enterocolitis’ OR ‘necrotising enterocolitis’/exp or ‘necrotising enterocolitis’) AND (ultrasonograph* OR sonograph* OR ultrasound*) AND [English]/lim AND [embase]/lim NOT [medline]/lim

Online Resource 1**:** Search strategy for Pubmed, CINAHL, and Embase.

	Portal gas		no Portal gas			Risk Difference
Study or Subgroup	Events	Total	Events	Total	Weight	M-H, Random, 95% CI
**1.1.1 Surgery**						
Bomelburg 1992	5	10	6	17	9.0%	0.15 [-0.24, 0.53]
He 2016	10	32	44	206	12.1%	0.10 [-0.07, 0.27]
Lindley 1986	2	5	1	10	7.8%	0.30 [-0.17, 0.77]
Yikilmaz 2014	2	5	4	21	7.9%	0.21 [-0.25, 0.67]
**Subtotal (95% CI)**		**52**		**254**	**36.7%**	**0.13 [-0.01, 0.27]**
Total events	19		55			
Heterogeneity: Tau² = 0.00; Chi² = 0.75, df = 3 (P = 0.86); I² = 0%						
Test for overall effect: Z = 1.87 (P = 0.06)						
**1.1.2 Surgery or Death**						
Garbi-Goutel 2014	7	13	19	82	10.5%	0.31 [0.02, 0.59]
Muchantef 2013	1	2	32	53	5.1%	-0.10 [-0.81, 0.60]
Palleri 2017	3	3	8	22	9.0%	0.64 [0.26, 1.02]
Prithviraj 2015	16	24	0	36	11.9%	0.67 [0.48, 0.86]
Silva 2007	4	5	18	35	8.9%	0.29 [-0.10, 0.67]
Staryszak 2015	0	1	8	8	5.9%	-1.00 [-1.62, -0.38]
Wang 2016	15	35	40	123	11.9%	0.10 [-0.08, 0.29]
**Subtotal (95% CI)**		**83**		**359**	**63.3%**	**0.20 [-0.11, 0.51]**
Total events	46		125			
Heterogeneity: Tau² = 0.14; Chi² = 40.51, df = 6 (P < 0.00001); I² = 85%						
Test for overall effect: Z = 1.26 (P = 0.21)						
**Total (95% CI)**		**135**		**613**	**100.0%**	**0.21 [0.00, 0.41]**
Total events	65		180			
Heterogeneity: Tau² = 0.08; Chi² = 45.85, df = 10 (P < 0.00001); I² = 78%						
Test for overall effect: Z = 2.00 (P = 0.05)						
Test for subgroup differences: Chi² = 0.14, df = 1 (P = 0.70), I² = 0%						

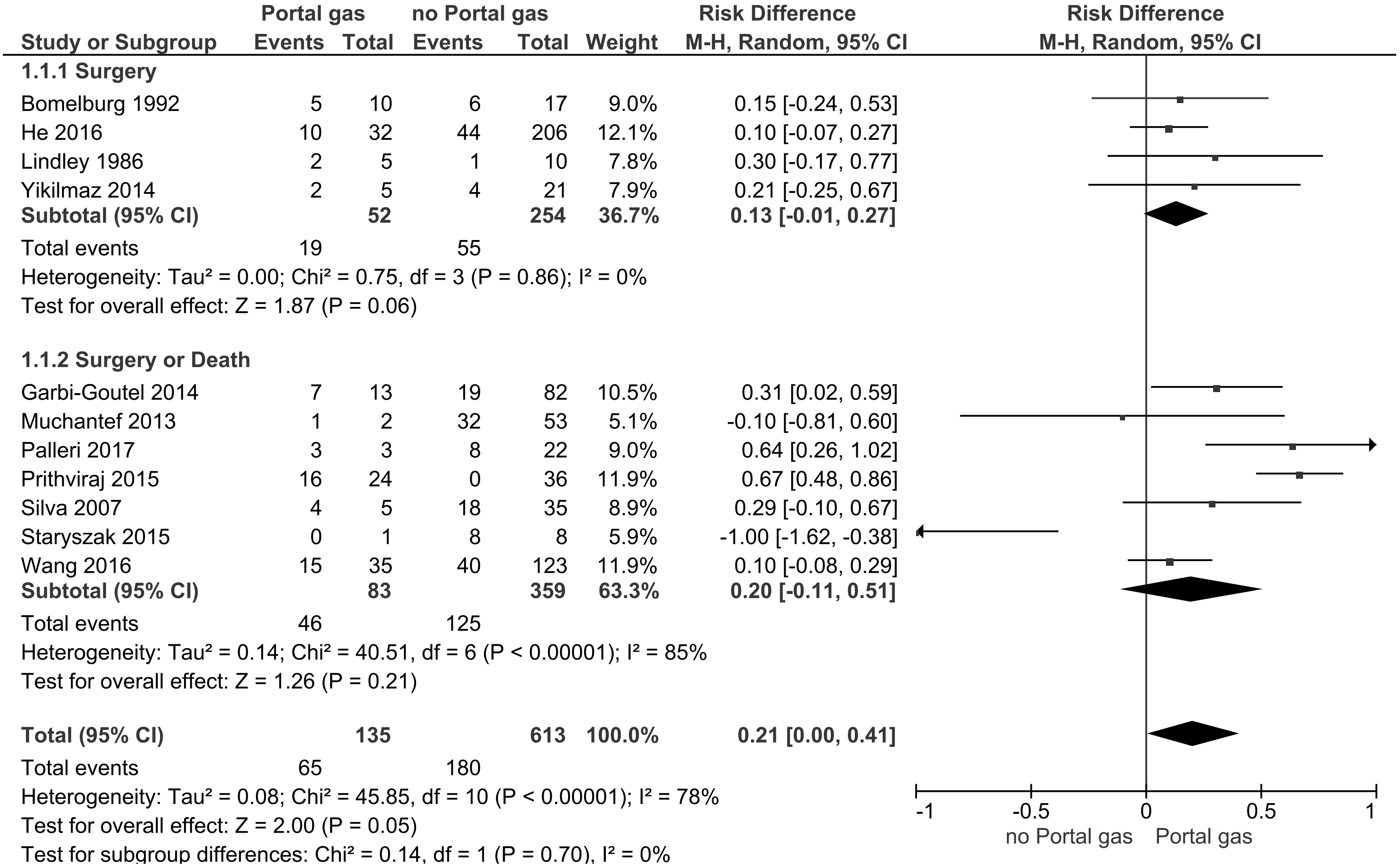

Online Resource 2: Forest plots for association of portal venous gas with surgery and surgery or death. Event refers to either surgery or surgery or death. Odds ratios (95% confidence intervals) for each study are denoted by boxes (lines and arrows). Combined odds ratio estimate for all studies is represented by the diamond. Tau-squared (Tau2) >1, Cochran Q (Chi2) P<0.1, and inconsistency (I2) ≥50% suggests substantial statistical heterogeneity. Z score with P<0.05 indicates test for overall effect was statistically significant. CI = confidence interval. M-H = Mantel-Haenszel test.

Study or Subgroup	Pneumatosis Events	Pneumatosis Total	no Pneumatosis Events	no Pneumatosis Total	Weight	Odds Ratio M-H, Random, 95% CI
**2.1.1 Surgery**						
Bomelburg 1992	6	14	2	9	8.3%	2.63 [0.39, 17.46]
He 2016	8	24	46	214	18.1%	1.83 [0.74, 4.53]
Yikilmaz 2014	8	16	0	10	4.0%	21.00 [1.05, 418.48]
**Subtotal (95% CI)**		**54**		**233**	**30.3%**	**2.61 [0.93, 7.34]**
Total events	22		48			
Heterogeneity: Tau² = 0.22; Chi² = 2.52, df = 2 (P = 0.28); I² = 21%						
Test for overall effect: Z = 1.81 (P = 0.07)						
**2.1.2 Surgery or Death**						
Garbi-Goutel 2014	18	51	8	44	17.4%	2.45 [0.94, 6.39]
Muchantef 2013	6	7	27	48	6.7%	4.67 [0.52, 41.80]
Palleri 2017	7	19	4	6	8.0%	0.29 [0.04, 2.02]
Prithviraj 2015	16	31	0	29	4.3%	62.81 [3.53, 1118.73]
Silva 2007	14	21	8	19	13.3%	2.75 [0.76, 9.95]
Staryszak 2015	3	4	5	5	3.1%	0.21 [0.01, 6.82]
Wang 2016	8	18	47	140	16.9%	1.58 [0.59, 4.28]
**Subtotal (95% CI)**		**151**		**291**	**69.7%**	**2.07 [0.85, 5.05]**
Total events	72		99			
Heterogeneity: Tau² = 0.67; Chi² = 12.66, df = 6 (P = 0.05); I² = 53%						
Test for overall effect: Z = 1.60 (P = 0.11)						
**Total (95% CI)**		**205**		**524**	**100.0%**	**2.25 [1.18, 4.31]**
Total events	94		147			
Heterogeneity: Tau² = 0.39; Chi² = 15.21, df = 9 (P = 0.09); I² = 41%						
Test for overall effect: Z = 2.46 (P = 0.01)						
Test for subgroup differences: Chi² = 0.11, df = 1 (P = 0.74), I² = 0%						

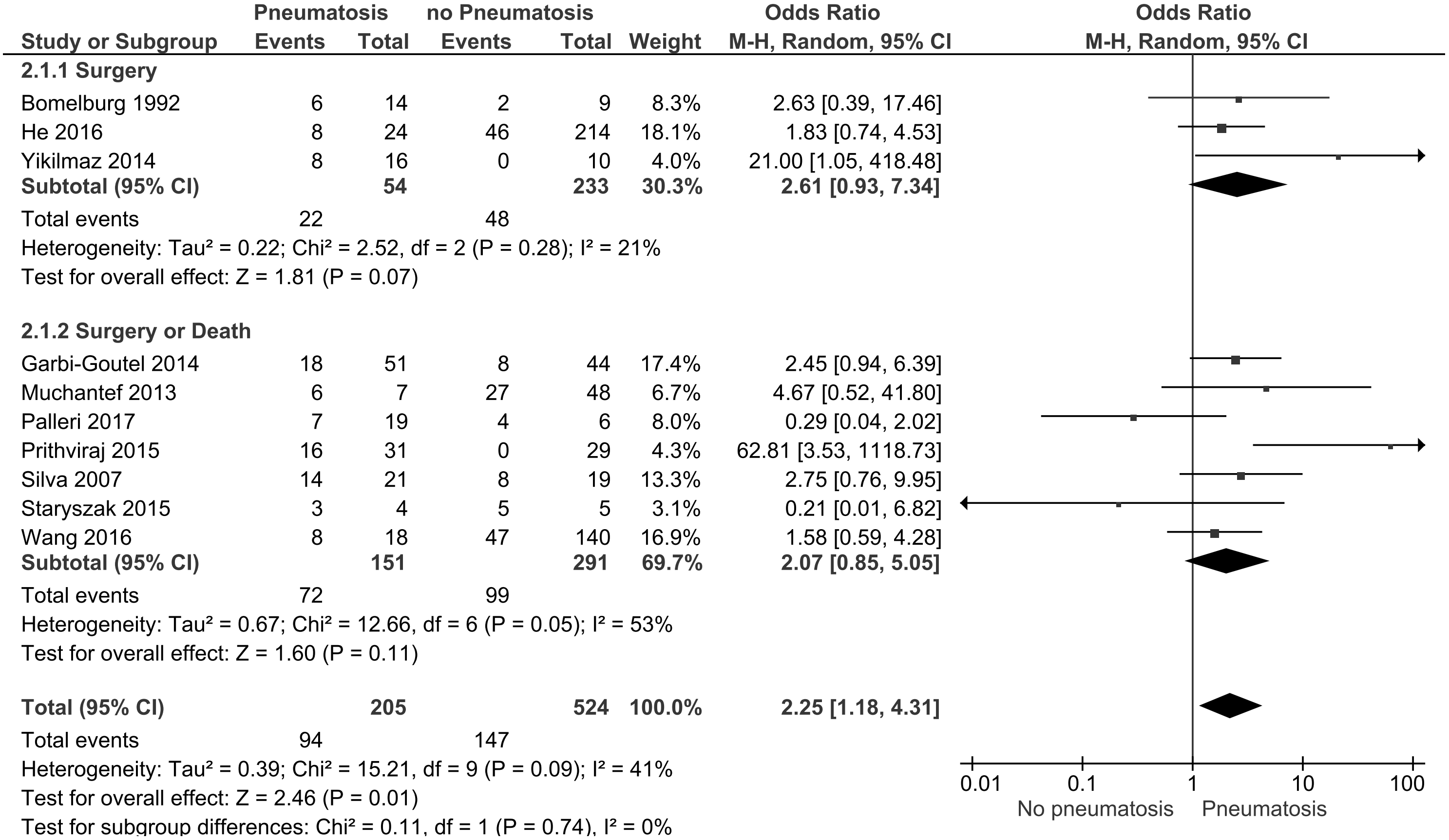

Online Resource 3: Forest plots for association of pneumatosis with surgery and surgery or death. See Online Resource 2 legend for an explanation of symbols and statistics.

	Free air		No free air			Odds Ratio
**Study or Subgroup**	**Events**	**Total**	**Events**	**Total**	**Weight**	**M-H, Random, 95% CI**
**3.1.1 Surgery or Death**						
Garbi-Goutel 2014	5	7	21	88	22.2%	7.98 [1.44, 44.17]
Muchantef 2013	7	8	26	47	19.7%	5.65 [0.64, 49.66]
Prithviraj 2015	10	10	6	50	15.7%	143.77 [7.49, 2757.85]
Silva 2007	6	6	16	34	15.7%	14.58 [0.76, 279.04]
Staryszak 2015	0	1	8	8	10.6%	0.02 [0.00, 1.43]
Wang 2016	13	13	42	145	16.2%	65.75 [3.82, 1131.24]
**Subtotal (95% CI)**		**45**		**372**	**100.0%**	**9.63 [1.65, 56.32]**
Total events	41		119			
Heterogeneity: Tau² = 2.90; Chi² = 13.32, df = 5 (P = 0.02); I² = 62%						
Test for overall effect: Z = 2.51 (P = 0.01)						
**Total (95% CI)**		**45**		**372**	**100.0%**	**9.63 [1.65, 56.32]**
Total events	41		119			
Heterogeneity: Tau² = 2.90; Chi² = 13.32, df = 5 (P = 0.02); I² = 62%						
Test for overall effect: Z = 2.51 (P = 0.01)						
Test for subgroup differences: Not applicable						

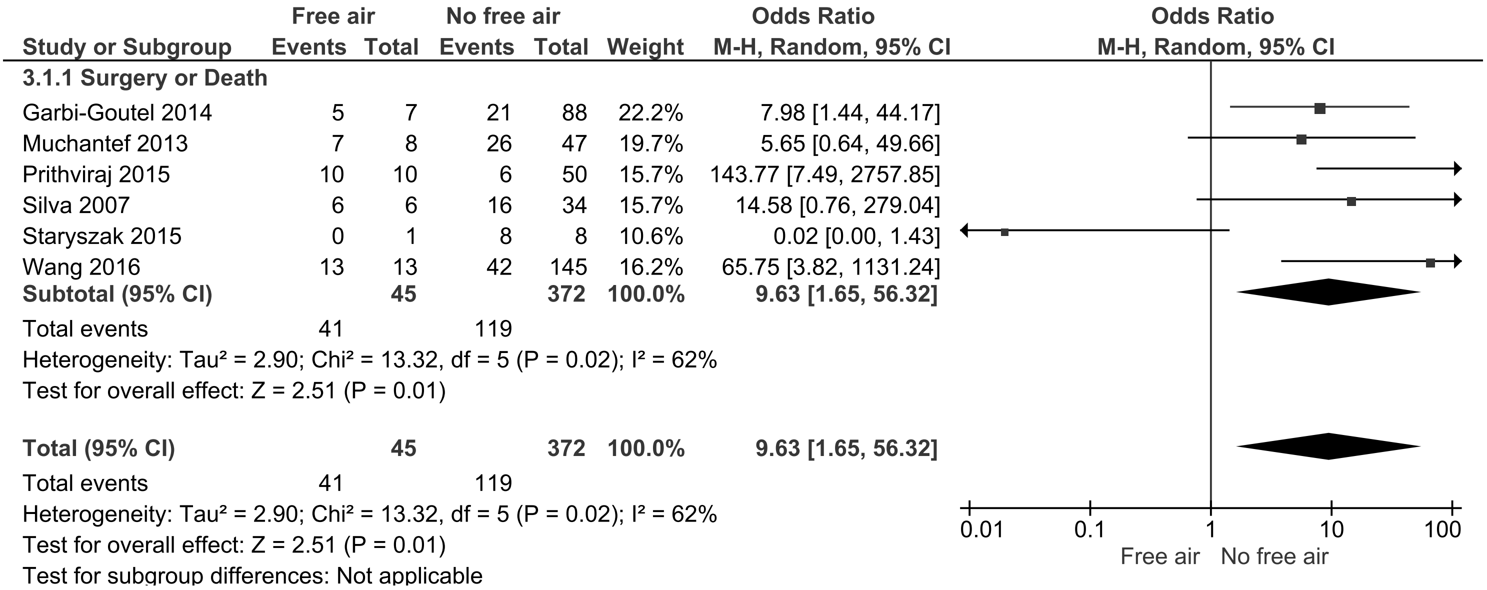

Online Resource 4: Forest plot for association of free air with surgery or death in NEC. See Online Resource 2 legend for an explanation of symbols and statistics.

Study or Subgroup	Wall thickness Events	Wall thickness Total	No wall thickness Events	No wall thickness Total	Weight	Odds Ratio M-H, Random, 95% CI
**5.1.2 Surgery or Death**						
Garbi-Goutel 2014	13	31	13	64	24.4%	2.83 [1.11, 7.24]
Muchantef 2013	23	30	10	25	15.8%	4.93 [1.54, 15.79]
Palleri 2017	3	5	8	20	5.4%	2.25 [0.30, 16.63]
Prithviraj 2015	16	43	0	17	2.6%	21.00 [1.18, 372.84]
Silva 2007	15	19	7	21	10.5%	7.50 [1.80, 31.28]
Staryszak 2015	8	8	0	1	1.2%	51.00 [0.70, 3710.31]
Wang 2016	23	42	32	116	40.1%	3.18 [1.53, 6.60]
**Subtotal (95% CI)**		**178**		**264**	**100.0%**	**3.86 [2.43, 6.14]**
Total events	101		70			
Heterogeneity: Tau² = 0.00; Chi² = 4.78, df = 6 (P = 0.57); I² = 0%						
Test for overall effect: Z = 5.71 (P < 0.00001)						
**Total (95% CI)**		**178**		**264**	**100.0%**	**3.86 [2.43, 6.14]**
Total events	101		70			
Heterogeneity: Tau² = 0.00; Chi² = 4.78, df = 6 (P = 0.57); I² = 0%						
Test for overall effect: Z = 5.71 (P < 0.00001)						
Test for subgroup differences: Not applicable						

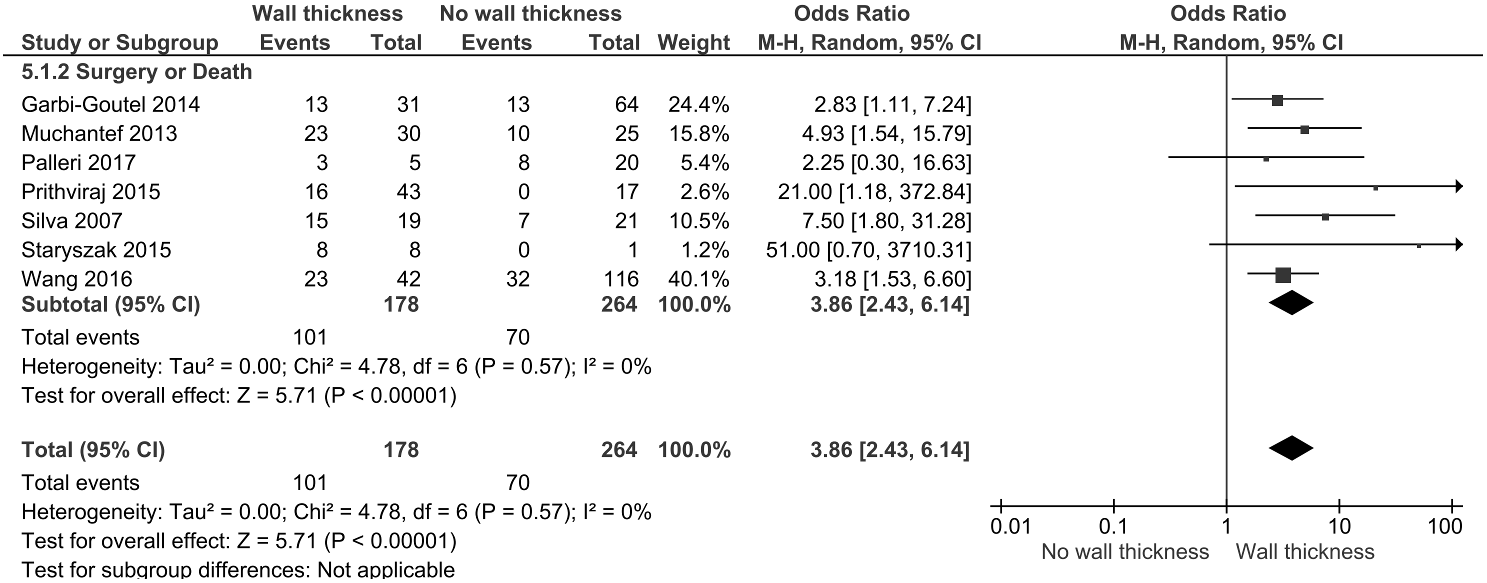

Online Resource 5: Forest plot for association of bowel wall thickening with surgery or death. See Online Resource 2 legend for an explanation of symbols and statistics.

Study or Subgroup	Wall thinness Events	Wall thinness Total	No wall thinness Events	No wall thinness Total	Weight	Odds Ratio M-H, Random, 95% CI
**5.2.2 Surgery or Death**						
Muchantef 2013	10	11	23	44	22.0%	9.13 [1.08, 77.53]
Palleri 2017	5	11	6	14	27.4%	1.11 [0.23, 5.45]
Prithviraj 2015	9	10	7	50	21.4%	55.29 [6.03, 506.52]
Silva 2007	15	19	7	21	29.2%	7.50 [1.80, 31.28]
Staryszak 2015	0	0	8	9		Not estimable
**Subtotal (95% CI)**		**51**		**138**	**100.0%**	**7.11 [1.56, 32.29]**
Total events	39		51			
Heterogeneity: Tau² = 1.52; Chi² = 8.45, df = 3 (P = 0.04); I² = 64%						
Test for overall effect: Z = 2.54 (P = 0.01)						
**Total (95% CI)**		**51**		**138**	**100.0%**	**7.11 [1.56, 32.29]**
Total events	39		51			
Heterogeneity: Tau² = 1.52; Chi² = 8.45, df = 3 (P = 0.04); I² = 64%						
Test for overall effect: Z = 2.54 (P = 0.01)						
Test for subgroup differences: Not applicable						

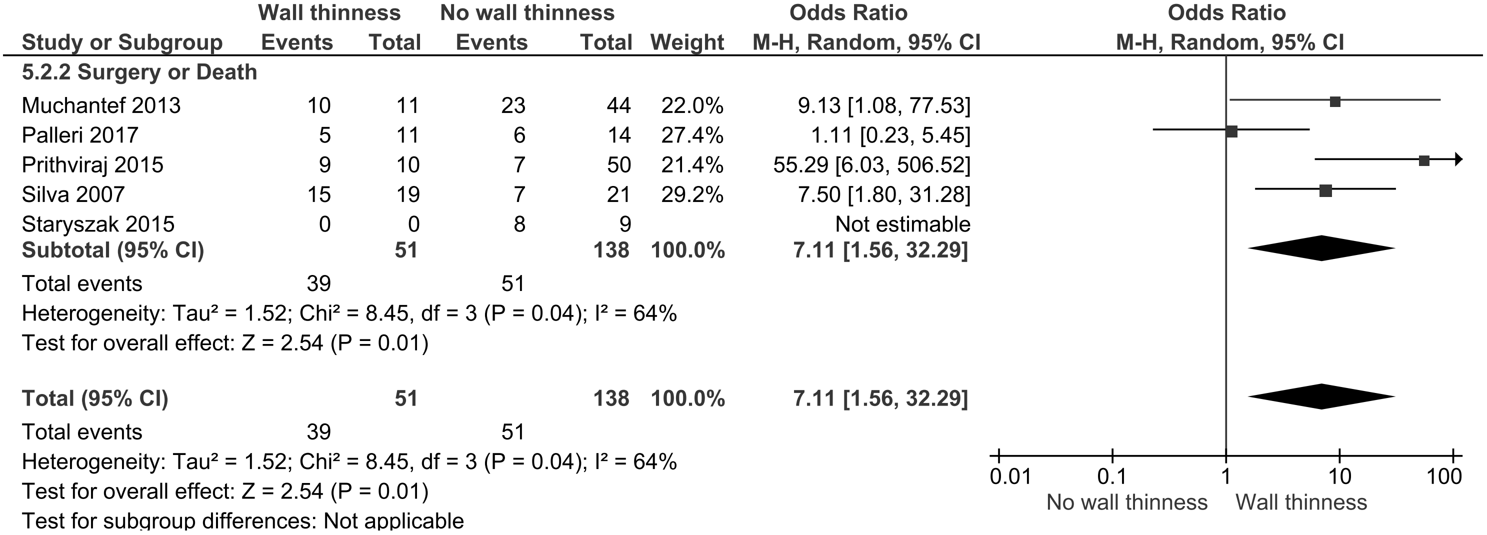

Online Resource 6: Forest plot for association of bowel wall thinning with surgery or death. See Online Resource 2 legend for an explanation of symbols and statistics.

Study or Subgroup	Echogenecity Events	Echogenecity Total	No echogenecity Events	No echogenecity Total	Weight	Odds Ratio M-H, Random, 95% CI	Odds Ratio M-H, Random, 95% CI
**4.1.2 Surgery or Death**							
Muchantef 2013	24	30	9	25	57.8%	7.11 [2.12, 23.88]	
Silva 2007	18	22	4	18	35.2%	15.75 [3.34, 74.35]	
Staryszak 2015	3	3	5	6	7.0%	1.91 [0.06, 61.34]	
**Subtotal (95% CI)**		**55**		**49**	**100.0%**	**8.58 [3.42, 21.53]**	
Total events	45		18				
Heterogeneity: Tau² = 0.00; Chi² = 1.40, df = 2 (P = 0.50); I² = 0%							
Test for overall effect: Z = 4.57 (P < 0.00001)							
**Total (95% CI)**		**55**		**49**	**100.0%**	**8.58 [3.42, 21.53]**	
Total events	45		18				
Heterogeneity: Tau² = 0.00; Chi² = 1.40, df = 2 (P = 0.50); I² = 0%							
Test for overall effect: Z = 4.57 (P < 0.00001)							
Test for subgroup differences: Not applicable							

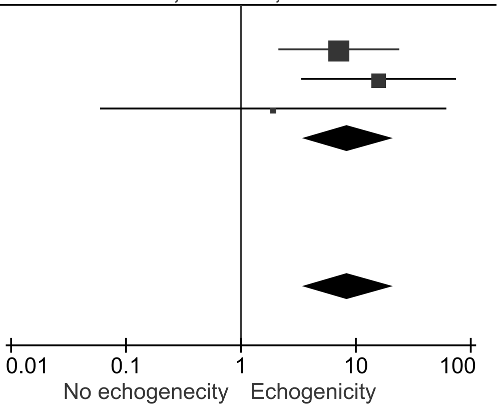

Online Resource 7: Forest plot for association of increased bowel wall echogenicity with surgery or death. See Online Resource 2 legend for an explanation of symbols and statistics.

Study or Subgroup	Absent peristalsis Events	Absent peristalsis Total	No absent peristalsis Events	No absent peristalsis Total	Weight	Odds Ratio M-H, Random, 95% CI
**10.1.2 Surgery or Death**						
Muchantef 2013	19	21	14	34	25.6%	13.57 [2.72, 67.84]
Palleri 2017	7	11	4	14	25.1%	4.38 [0.81, 23.69]
Prithviraj 2015	14	15	2	45	20.3%	301.00 [25.33, 3576.60]
Wang 2016	9	19	46	139	29.1%	1.82 [0.69, 4.79]
**Subtotal (95% CI)**		**66**		**232**	**100.0%**	**10.68 [1.65, 69.02]**
Total events	49		66			
Heterogeneity: Tau² = 2.87; Chi² = 16.65, df = 3 (P = 0.0008); I² = 82%						
Test for overall effect: Z = 2.49 (P = 0.01)						
**Total (95% CI)**		**66**		**232**	**100.0%**	**10.68 [1.65, 69.02]**
Total events	49		66			
Heterogeneity: Tau² = 2.87; Chi² = 16.65, df = 3 (P = 0.0008); I² = 82%						
Test for overall effect: Z = 2.49 (P = 0.01)						
Test for subgroup differences: Not applicable						

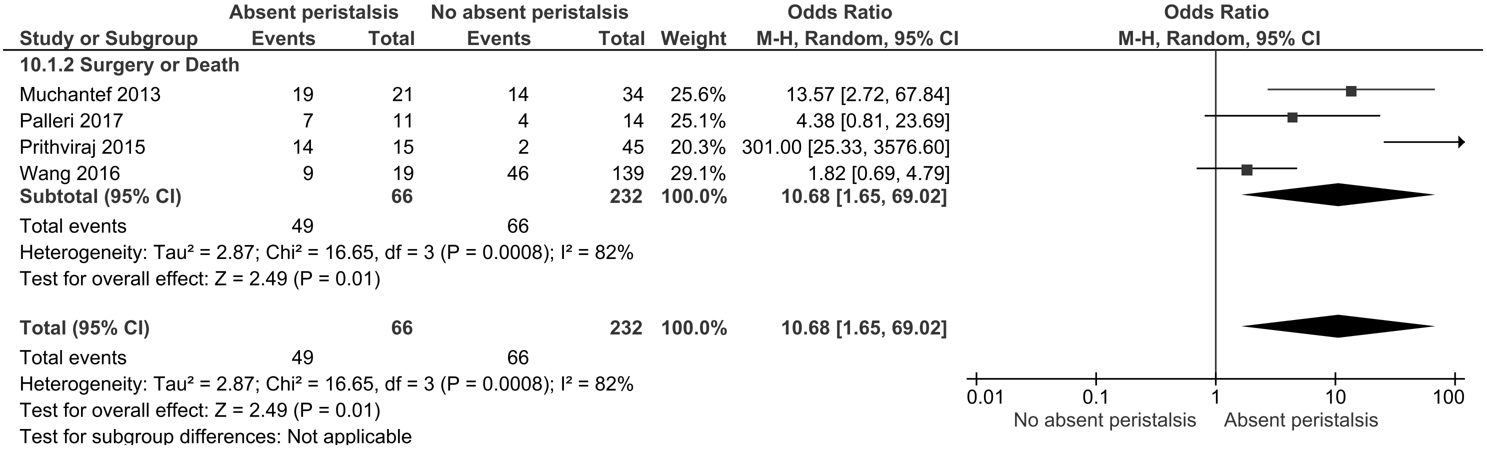

Online Resource 8: Forest plot for association of absent peristalsis with surgery or death. See Online Resource 2 legend for an explanation of symbols and statistics.

Study or Subgroup	Increased perfusion Events	Increased perfusion Total	No increased perfusion Events	No increased perfusion Total	Weight	Odds Ratio M-H, Random, 95% CI	Odds Ratio M-H, Random, 95% CI
**9.1.2 Surgery or Death**							
Muchantef 2013	17	26	16	29	31.1%	1.53 [0.52, 4.57]	
Palleri 2017	8	16	3	9	24.9%	2.00 [0.37, 10.92]	
Prithviraj 2015	16	27	0	33	15.1%	96.13 [5.33, 1733.40]	
Silva 2007	14	26	8	14	28.9%	0.88 [0.24, 3.24]	
**Subtotal (95% CI)**		**95**		**85**	**100.0%**	**2.60 [0.61, 11.13]**	
Total events	55		27				
Heterogeneity: Tau² = 1.45; Chi² = 10.07, df = 3 (P = 0.02); I² = 70%							
Test for overall effect: Z = 1.29 (P = 0.20)							
**Total (95% CI)**		**95**		**85**	**100.0%**	**2.60 [0.61, 11.13]**	
Total events	55		27				
Heterogeneity: Tau² = 1.45; Chi² = 10.07, df = 3 (P = 0.02); I² = 70%							
Test for overall effect: Z = 1.29 (P = 0.20)							
Test for subgroup differences: Not applicable							

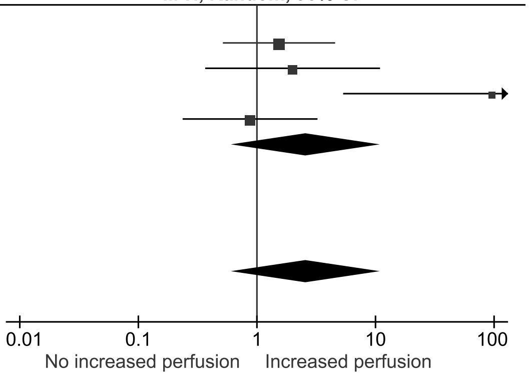

Online Resource 9: Forest plot for association of increased bowel perfusion with surgery or death. See Online Resource 2 legend for an explanation of symbols and statistics.

Study or Subgroup	Absent perfusion Events	Absent perfusion Total	No absent perfusion Events	No absent perfusion Total	Weight	Odds Ratio M-H, Random, 95% CI
**9.2.2 Surgery or Death**						
Muchantef 2013	11	12	22	43	32.9%	10.50 [1.24, 88.59]
Palleri 2017	3	3	8	22	15.8%	11.94 [0.55, 260.28]
Silva 2007	8	10	14	30	51.3%	4.57 [0.83, 25.21]
**Subtotal (95% CI)**		**25**		**95**	**100.0%**	**6.99 [2.06, 23.76]**
Total events	22		44			
Heterogeneity: Tau² = 0.00; Chi² = 0.50, df = 2 (P = 0.78); I² = 0%						
Test for overall effect: Z = 3.12 (P = 0.002)						
**Total (95% CI)**		**25**		**95**	**100.0%**	**6.99 [2.06, 23.76]**
Total events	22		44			
Heterogeneity: Tau² = 0.00; Chi² = 0.50, df = 2 (P = 0.78); I² = 0%						
Test for overall effect: Z = 3.12 (P = 0.002)						
Test for subgroup differences: Not applicable						

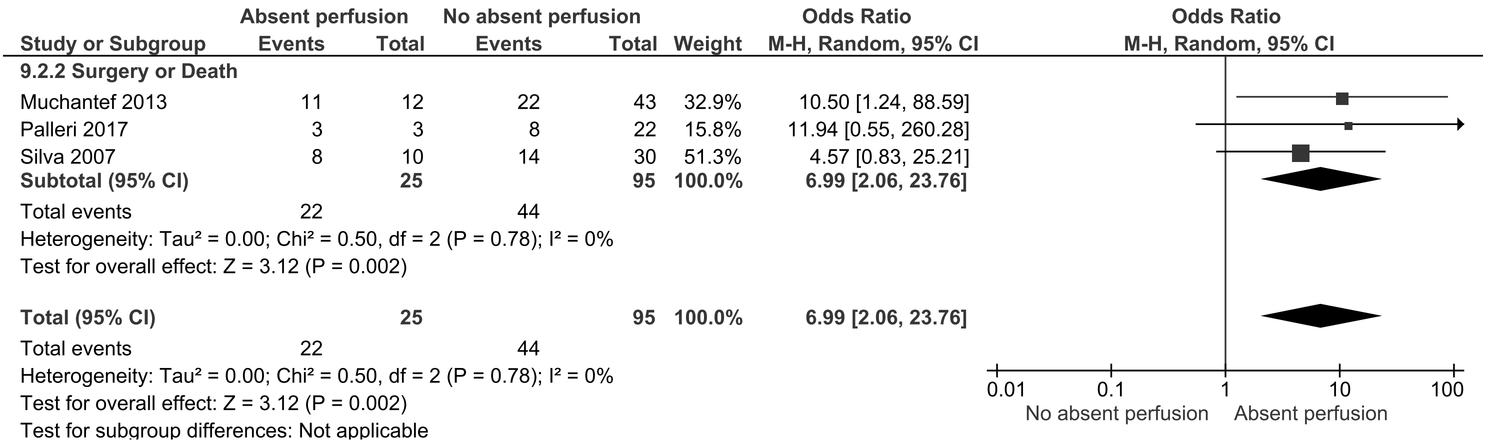

Online Resource 10: Forest plot for association of absent perfusion with surgery or death. See Online Resource 2 legend for an explanation of symbols and statistics.

	Simple ascites		No simple ascites			Odds Ratio
**Study or Subgroup**	**Events**	**Total**	**Events**	**Total**	**Weight**	**M-H, Random, 95% CI**
**6.1.2 Surgery or Death**						
Muchantef 2013	5	15	28	40	23.2%	0.21 [0.06, 0.76]
Palleri 2017	6	11	5	14	22.3%	2.16 [0.43, 10.84]
Prithviraj 2015	13	13	3	47	17.8%	343.29 [16.67, 7070.48]
Silva 2007	1	6	21	34	20.3%	0.12 [0.01, 1.18]
Staryszak 2015	4	4	4	5	16.4%	3.00 [0.09, 95.17]
**Subtotal (95% CI)**		**49**		**140**	**100.0%**	**1.84 [0.17, 20.41]**
Total events	29		61			
Heterogeneity: Tau² = 6.09; Chi² = 26.71, df = 4 (P < 0.0001); I² = 85%						
Test for overall effect: Z = 0.50 (P = 0.62)						
**Total (95% CI)**		**49**		**140**	**100.0%**	**1.84 [0.17, 20.41]**
Total events	29		61			
Heterogeneity: Tau² = 6.09; Chi² = 26.71, df = 4 (P < 0.0001); I² = 85%						
Test for overall effect: Z = 0.50 (P = 0.62)						
Test for subgroup differences: Not applicable						

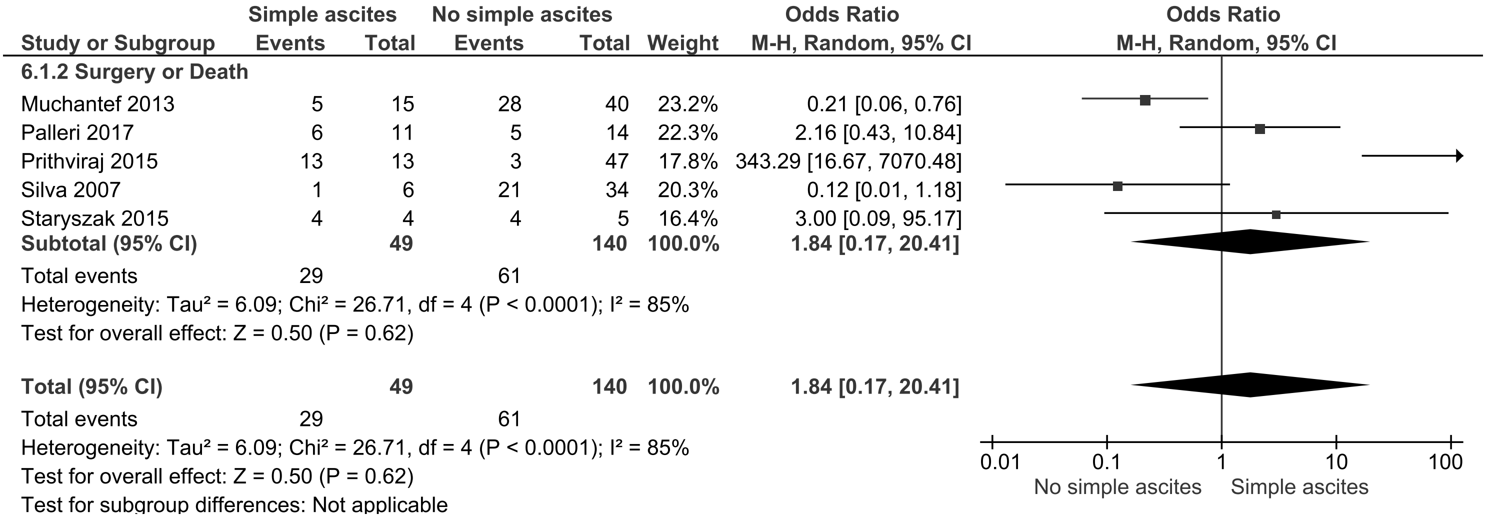

Online Resource 11: Forest plot for association of simple ascites with surgery or death. See Online Resource 2 legend for an explanation of symbols and statistics.

Study or Subgroup	Complex ascites Events	Complex ascites Total	No complex ascites Events	No complex ascites Total	Weight	Odds Ratio M-H, Random, 95% CI
**6.2.2 Surgery or Death**						
Muchantef 2013	25	28	8	27	45.4%	19.79 [4.62, 84.78]
Silva 2007	17	22	5	18	46.7%	8.84 [2.11, 37.11]
Staryszak 2015	3	3	5	6	8.0%	1.91 [0.06, 61.34]
**Subtotal (95% CI)**		**53**		**51**	**100.0%**	**11.28 [4.23, 30.04]**
Total events	45		18			
Heterogeneity: Tau² = 0.00; Chi² = 1.69, df = 2 (P = 0.43); I² = 0%						
Test for overall effect: Z = 4.85 (P < 0.00001)						
**Total (95% CI)**		**53**		**51**	**100.0%**	**11.28 [4.23, 30.04]**
Total events	45		18			
Heterogeneity: Tau² = 0.00; Chi² = 1.69, df = 2 (P = 0.43); I² = 0%						
Test for overall effect: Z = 4.85 (P < 0.00001)						
Test for subgroup differences: Not applicable						

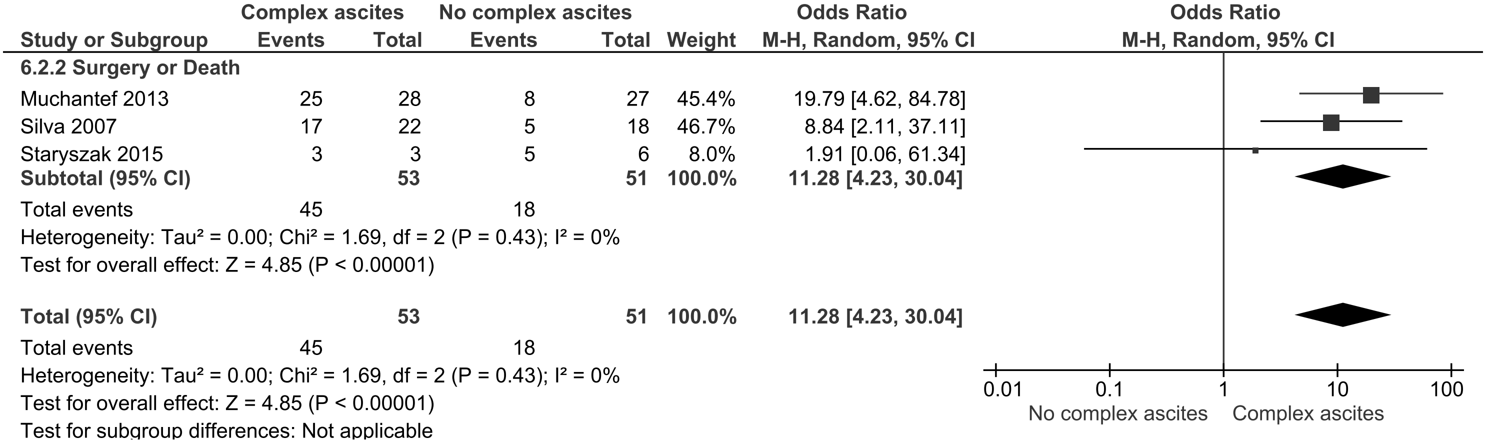

Online Resource 12: Forest plot for association of complex ascites with surgery or death. See Online Resource 2 legend for an explanation of symbols and statistics.

	Focal fluid collection		No focal fluid collection			Odds Ratio
**Study or Subgroup**	**Events**	**Total**	**Events**	**Total**	**Weight**	**M-H, Random, 95% CI**
**7.1.2 Surgery or Death**						
Garbi-Goutel 2014	5	9	21	86	27.6%	3.87 [0.95, 15.75]
Muchantef 2013	12	13	21	42	22.5%	12.00 [1.43, 100.75]
Palleri 2017	7	8	4	17	20.8%	22.75 [2.11, 244.87]
Prithviraj 2015	16	16	0	44	12.4%	2937.00 [55.96, 154132.85]
Silva 2007	3	3	19	37	16.8%	6.64 [0.32, 137.55]
**Subtotal (95% CI)**		**49**		**226**	**100.0%**	**17.92 [3.11, 103.31]**
Total events	43		65			
Heterogeneity: Tau² = 2.38; Chi² = 10.85, df = 4 (P = 0.03); I² = 63%						
Test for overall effect: Z = 3.23 (P = 0.001)						
**Total (95% CI)**		**49**		**226**	**100.0%**	**17.92 [3.11, 103.31]**
Total events	43		65			
Heterogeneity: Tau² = 2.38; Chi² = 10.85, df = 4 (P = 0.03); I² = 63%						
Test for overall effect: Z = 3.23 (P = 0.001)						
Test for subgroup differences: Not applicable						

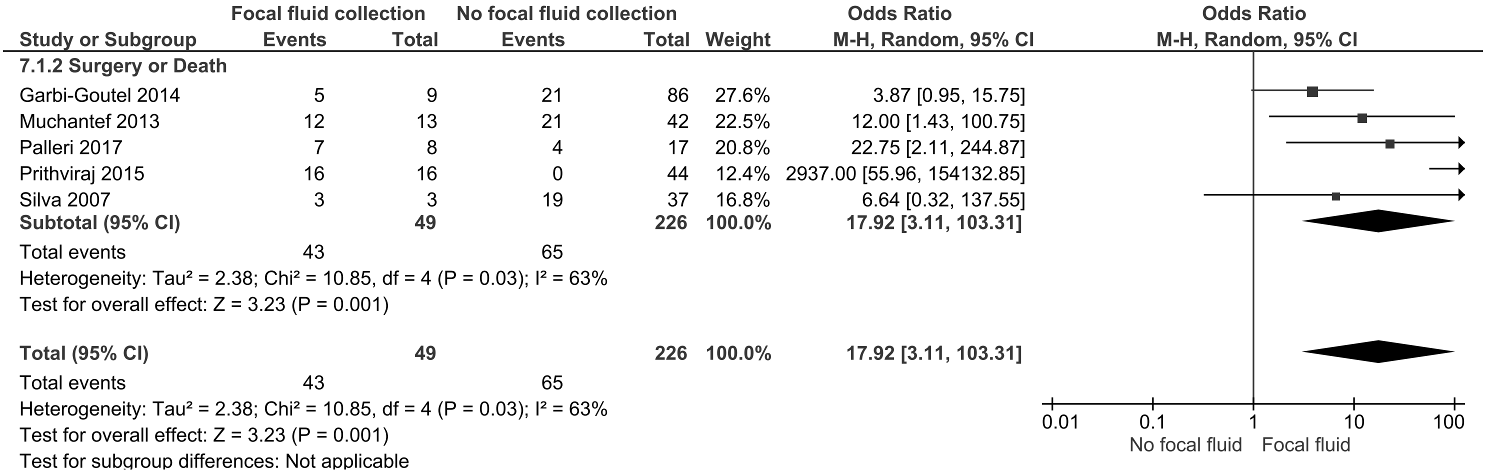

Online Resource 13: Forest plot for association of focal fluid collection with surgery or death. See Online Resource 2 legend for an explanation of symbols and statistics.

Study or Subgroup	Dilated bowel Events	Dilated bowel Total	No dilated bowel Events	No dilated bowel Total	Weight	Odds Ratio M-H, Random, 95% CI
**8.1.1 Surgery or Death**						
Muchantef 2013	3	3	30	52	4.8%	5.16 [0.25, 105.06]
Staryszak 2015	4	5	4	4	3.6%	0.33 [0.01, 10.57]
Wang 2016	34	65	21	93	91.6%	3.76 [1.89, 7.48]
**Subtotal (95% CI)**		**73**		**149**	**100.0%**	**3.50 [1.81, 6.75]**
Total events	41		55			
Heterogeneity: Tau² = 0.00; Chi² = 1.89, df = 2 (P = 0.39); I² = 0%						
Test for overall effect: Z = 3.73 (P = 0.0002)						
**Total (95% CI)**		**73**		**149**	**100.0%**	**3.50 [1.81, 6.75]**
Total events	41		55			
Heterogeneity: Tau² = 0.00; Chi² = 1.89, df = 2 (P = 0.39); I² = 0%						
Test for overall effect: Z = 3.73 (P = 0.0002)						
Test for subgroup differences: Not applicable						

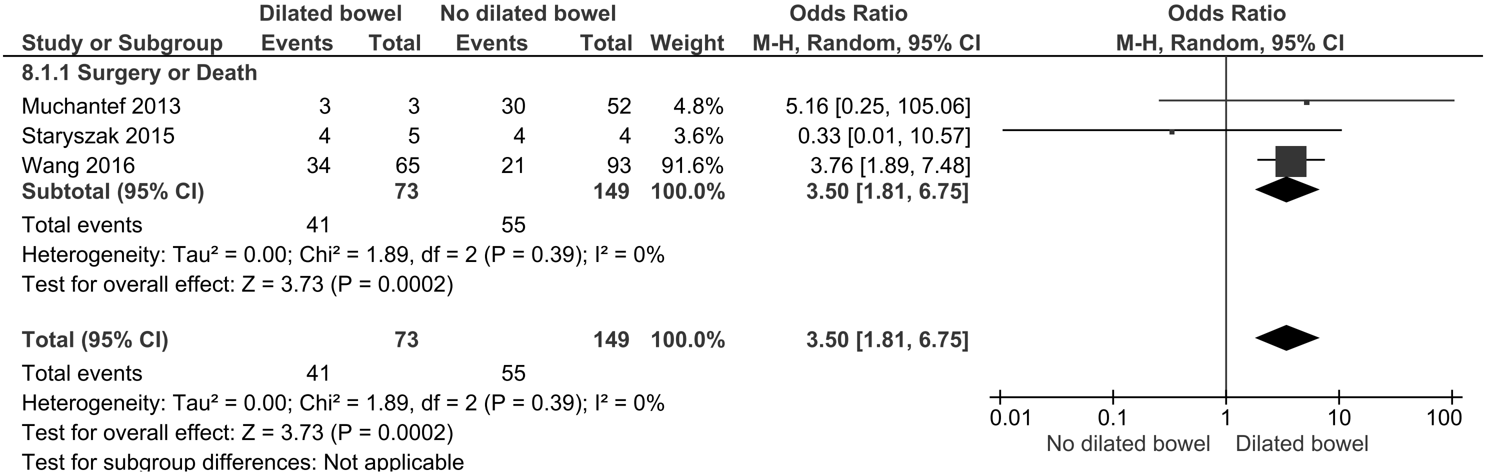

Online Resource 14: Forest plots for association of dilated bowel with surgery or death. See Online Resource 2 legend for an explanation of symbols and statistics.
